# Cancer drug resistance: redox resetting renders a way

**DOI:** 10.18632/oncotarget.8600

**Published:** 2016-04-05

**Authors:** Yuan Liu, Qifu Li, Li Zhou, Na Xie, Edouard C. Nice, Haiyuan Zhang, Canhua Huang, Yunlong Lei

**Affiliations:** ^1^ State Key Laboratory for Biotherapy and Cancer Center, West China Hospital, Sichuan University, and Collaborative Innovation Center of Biotherapy, Chengdu, P. R. China; ^2^ Department of Neurology, The Affiliated Hospital of Hainan Medical College, Haikou, Hainan, P. R. China; ^3^ Department of Biochemistry and Molecular Biology, Monash University, Clayton, Victoria, Australia; ^4^ Department of Biochemistry and Molecular Biology, and Molecular Medicine and Cancer Research Center, Chongqing Medical University, Chongqing, P. R. China

**Keywords:** drug resistance, cancer therapy, oxidative stress, redox modifications, drug efflux

## Abstract

Disruption of redox homeostasis is a crucial factor in the development of drug resistance, which is a major problem facing current cancer treatment. Compared with normal cells, tumor cells generally exhibit higher levels of reactive oxygen species (ROS), which can promote tumor progression and development. Upon drug treatment, some tumor cells can undergo a process of *‘Redox Resetting’* to acquire a new redox balance with higher levels of ROS accumulation and stronger antioxidant systems. Evidence has accumulated showing that the *‘Redox Resetting’* enables cancer cells to become resistant to anticancer drugs by multiple mechanisms, including increased rates of drug efflux, altered drug metabolism and drug targets, activated prosurvival pathways and inefficient induction of cell death. In this article, we provide insight into the role of ‘*Redox Resetting*’ on the emergence of drug resistance that may contribute to pharmacological modulation of resistance.

## INTRODUCTION

**Figure 1:**
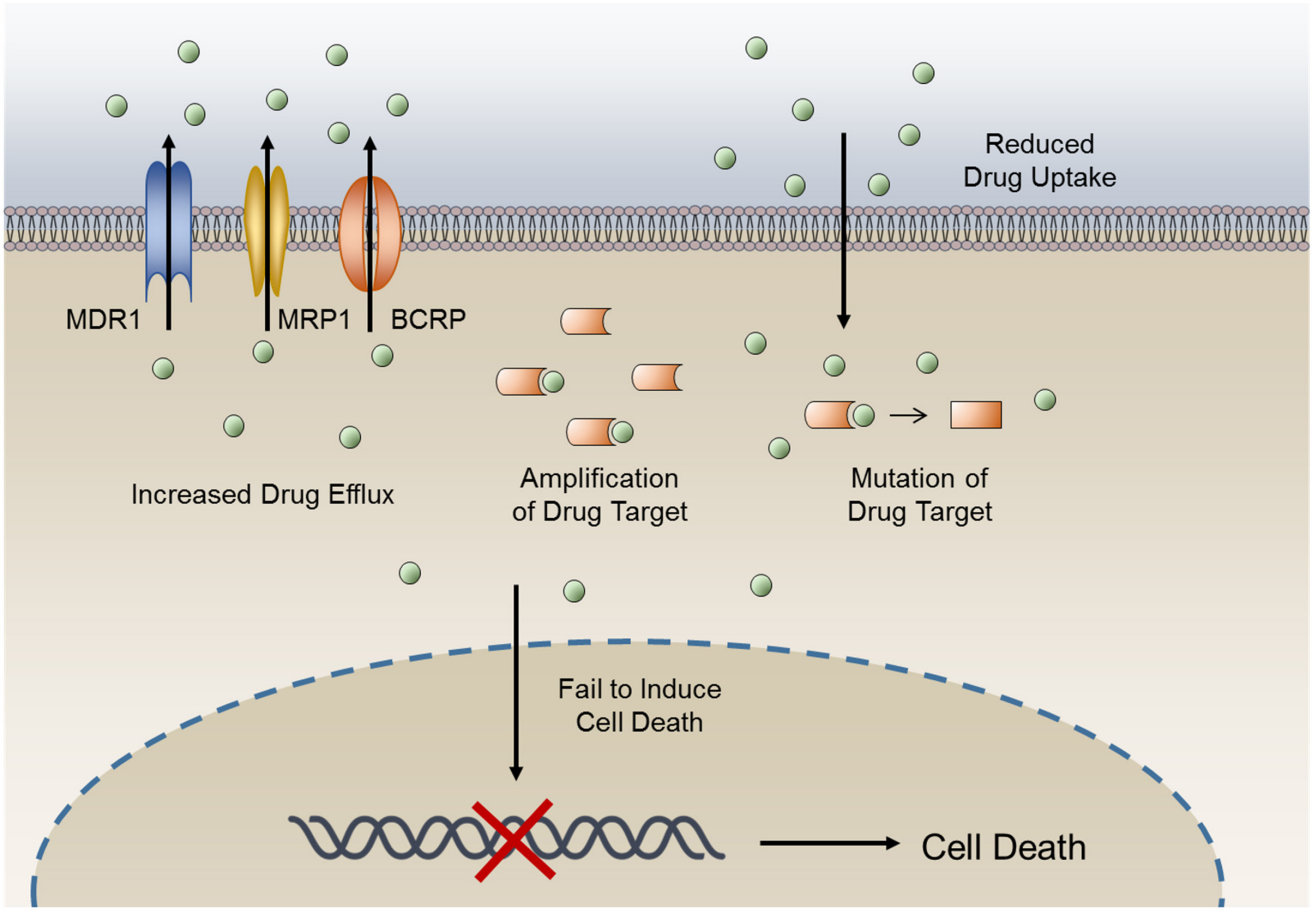
General mechanisms of cancer drug resistance The anticancer activity of a drug can be limited by reduced drug influx or increased drug efflux, changes in expression levels of drug target, mutation of drug target, and a lack of cell death induction.

Development of drug resistance is an important factor in the failure of anticancer therapeutic treatments [1]. Such resistance results from a variety of factors including individual variations in patients and somatic cell genetic differences in tumors. The ability to evade medicinal drugs is intrinsic to cancer cells. Reasons for acquisition of anticancer drug resistance include enhanced expression of transporters that increases anticancer drugs efflux, alterations in drug metabolism, mutations of drug targets and the activation of survival or inactivation of downstream death signaling pathways (Figure 1) [1]. Studies on cancer drug resistance mechanisms have yielded valuable information on how to circumvent resistance to improve cancer chemotherapy [1–3].

**Figure 2:**
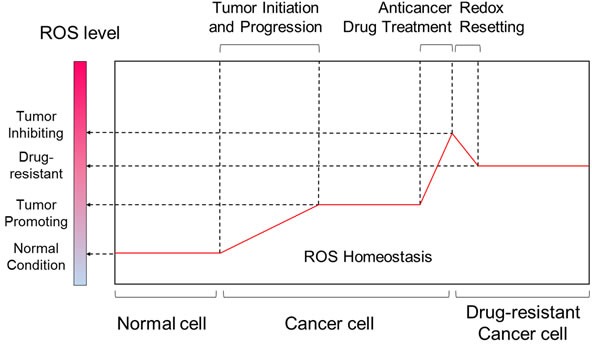
Comparisons of ROS level between different stages of tumor progression and tumor drug-resistance While in normal cells ROS generation and antioxidants are in balance, increased ROS levels are hallmarks of cancer cells. Marked increase in ROS can be achieved by chemotherapeutic agents, resulting in irreparable cellular damages and cancer cell death. However, some cancer cells can develop drug resistance by redox resetting.

Reactive oxygen species (ROS) are chemical oxygen species with reactive properties, which comprise hydrogen peroxide (H_2_O_2_), the hydroxyl radical (•OH), superoxide(O_2_^−^) and singlet oxygen (^1^O_2_) [4]. Under physiological conditions, cells are capable of maintaining a balance between cellular oxidants and antioxidants, called redox homeostasis. Submicromolar levels of ROS act as second messengers to regulate cell proliferation, cell death, and other cellular processes [5]. Excessive levels of ROS induce oxidative stress that leads to various pathological states, including aging, neurological disorders, and cancer [6]. In general, most tumors exhibit higher levels of ROS than normal tissues, thus promoting tumor progression and development [5]. Moreover, oxidative stress controls the efficacy of cancer treatments in multiple ways, including chemosensitivity, apoptosis, angiogenesis, metastasis and inflammatory responses [6]. However, when ROS concentrations become extremely high, they lead to tumor cell death [7]. Thus, a variety of drugs with direct or indirect effects on ROS induction have been used for effective cancer therapies (Table 1). Nonetheless, some tumor cells can overcome drug-induced oxidative stress by enhancing their antioxidant systems, with the outcome that a new redox balance with a more higher ROS level is established, the process of ‘*Redox Resetting*’ (Figure 2). Such drug-induced redox resetting has recently been shown to result in drug resistance. For example, increased levels of reduced glutathione lead to elevated chemotherapeutic drug resistance in numerous cancers [8, 9].

**Table 1:** Roles of anticancer treatments in regulating ROS levels

Name	Mechanism of action	Effects on ROS	Cancer types	Refs
Ionizing radiation	Photons or particles affect chemical bonds and produce highly ROS, which cause damage to DNA and other cellular components	Increases ROS production	Different types of cancer	[160]
Methotrexate	Triggers ROS associated cell apoptosis	Increases ROS production	Different types of cancer	[161]
Mitoxantrone	Triggers cell membrane scrambling	Significant increases of ROS formation	Different types of cancer	[162]
Tamoxifen	Promotes cancer cell senescence	Promotes ROS generation	Breast, colon cancer	[163]
Cisplatin	Generation of nuclear DNA adducts	Induces a mitochondrial-dependent ROS generation	Different types of cancer	[164]
Paclitaxel (Taxol)	Inhibitor of cell division	Increases ROS production	Different types of cancer	[165]
Adriamycin	Reduces cell viability through initiating cell apoptosis and strong G2/M phase cell cycle arrest	Increases ROS production	Different types of cancer	[166]
Imatinib	Protein tyrosine kinase inhibitor that induce apoptosis	Increases ROS production	Different types of cancer	[167]
Camptothecin	Quinolone alkaloid that induces cytotoxicity	Increases ROS production	Different types of cancer	[168]
Flavopiridol	Semisynthetic flavonoid that inhibits cyclin-dependent kinases	Increases ROS production	Leukemia	[169]
6-thioguanine	UVA photosensitizer	Increases ROS production	Skin cancer	[170]
Procarbazine	Isolated DNA could be degraded by procarbazine in the presence of oxygen	Increases ROS production	Lymphoma, primary brain cancers	[171]
NOV-002	Glutathione disulphide mimetic	Alters intracellular GSSG/GSH ratio	Lung, breast and ovarian cancer	[172]
Sulphasalazine	Inhibitor of cysteine/glutamate transporter xCT	Reduces intracellular transport of cysteine required for GSH synthesis	Pancreatic and lung cancer	[173, 174]
L-asparaginase	Depletes glutamine	Reduces GSH	Leukemia, pancreatic cancer	[175, 176]
Buthionine sulphoximine (BSO)	Glutamate-cysteine ligase complex inhibitor	Inhibits *de novo* GSH synthesis	Ovarian and breast cancer, melanoma	[177, 178]
Carboplatin	Induction of cell cycle arrest	Induction of ROS owing to ER stress	Different types of cancer	[179]
Gefitinib	Selective epidermal growth factor receptor tyrosine kinase inhibitor	Activates FOXO3a and in turn reduces ROS	Different types of cancer	[180]
Irinotecan	Topoisomerases inhibitor	Causes oxidative stress	Different types of cancer	[181]
Etoposide	Selective Topo II α inhibitor	Increases ROS production	Neuroblastoma, breast cancer	[182]
Tunicamycin	Glycosylation inhibitor that causes protein accumulation in the ER	Triggers ER stress production	Leukemia	[183]
Thapsigargin	Sarco(endo)plasmic reticulum Ca^2+^ ATPase inhibitor that releases ER Ca^2+^ and stimulates Ca^2+^ influx	Triggers ER stress production	Leukemia	[183]
Chloroethylnitrosoureas	Alkylating agent that causes DNA damage	Increases ROS production	Melanoma tumors	[184]
Temozolomide	Alkylating agent	Increases ROS production	Brain cancer	[185]
Celecoxib	Inhibits cyclooxygenase 2 (COX2) activity but it also induces ER stress by causing leakage of calcium from the ER into the cytosol	Induction of ROS owing to ER stress	Colorectal cancer, myeloma, Burkitt's lymphoma and prostate cancer	[186]
Nelfinavir	Originally developed as HIV protease inhibitor but it also induces ER stress by an unknown mechanism	Induction of ROS owing to ER stress	HPV-transformed cervical carcinoma, head and neck cancer, pancreatic cancer, melanoma and glioma	[187]
Bortezomib	Proteasome inhibitor	Induces ROS owing to ER stress	Mantle cell lymphoma, multiple myeloma	[188, 189]
Anthracyclines (doxorubicin, daunorubicin or epirubicin)	Insert into the DNA of replicating cells and inhibit topoisomerase II, which prevents DNA and RNA synthesis.	Induce the generation of oxygen-derived free radicals through two main pathways: anon-enzymatic pathway that utilizes iron, and anenzymatic mechanism that involves the mitochondrial respiratory chain	Different types of cancer	[190]
17-allylaminogeldanamycin (17-AAG)	HSP90 inhibitor	Decrease protein homeostasis during oxidative stress by disrupting HSP90–client protein complexes and promoting the degradation of the client proteins	Breast cancer, non-small-cell lung cancer	[191]
Capecitabine	Prodrug that is enzymatically converted to 5-fluorouracil (5-FU) in the body	Decreases ROS production	Colorectal, breast, gastric, and oesophageal cancer	[192]
5-fluorouracil (5-FU)	Inhibits thymidylate synthetase and/or incorporates into RNA and DNA	Induces intracellular increase inO2·- levels	Colon cancer, rectum cancer, and head and neck cancer	[88]
Arsenic trioxide (As2O3)	Reacts with cysteine residues on crucial proteins	Inhibits mitochondrial respiratory function, thereby increasing free radical generation	Leukemia, myeloma	[193]
2-methoxyestradiol(2-ME)	Metabolite of estradiol-17β	Induces free radicals and loss of mitochondrial membrane potential	Prostate cancer, leukemia	[194]
N-(4 hydroxyphenyl)retinamide (4-HPR)	Synthetic retinoid derivative	Induces apoptosis through the production of ROS and mitochondrial disruption	Prostate cancer, breast cancer, neuroblastoma	[195]
PARP inhibitors	Inhibit the action of the enzyme PARP	Reduce the capacity to repair ROS-induced DNA damage	Breast cancer	[196]
Erastin	Down regulates mitochondrial VDACs and cysteine redox shuttle	Alters the mitochondrial membrane permeability and blocks GSH regeneration	RAS^V12^-expressing tumor cells	[197, 198]

Redox resetting has been implicated in drug resistance at multiple levels, including elevated drug efflux, altered drug metabolism and mutated drug targets [10, 11]. In addition, ROS-induced activation of survival signaling pathways and inactivation of downstream death signaling pathways can lead to drug resistance (Figure 1) [1, 12, 13]. Here, we focus on the effects of redox resetting on drug resistance mechanisms and on current research efforts to reveal the detailed mechanisms of resistance to cancer therapies.

## INCREASED RATES OF DRUG EFFLUX

Drug export from cells is a primary cause of the cellular resistance to anticancer drugs and poses a significant threat to clinical tumor therapy. Several cell membrane transporter proteins have been implicated in drug resistance to commonly used chemotherapeutics by promoting drug efflux [1]. Among them, the ATP-binding cassette (ABC) transporter family is the most notable. There are 49 members of the ABC transporter family, but only multi-drug resistance protein 1 (MDR1), MDR-associated protein 1 (MRP1) and breast cancer resistance protein (BCRP) have been studied extensively in relation to multidrug resistance (MDR) [10]. All three transporters have broad substrate specificity and promote the efflux of various hydrophobic cancer chemotherapeutics such as topoisomerase inhibitors, taxanes, and antimetabolites [14]. Here, we summarize the effects of redox reactions and redox signals on these three drug efflux transporters.

### Redox reactions promote conformational changes of the transporters

**Figure 3:**
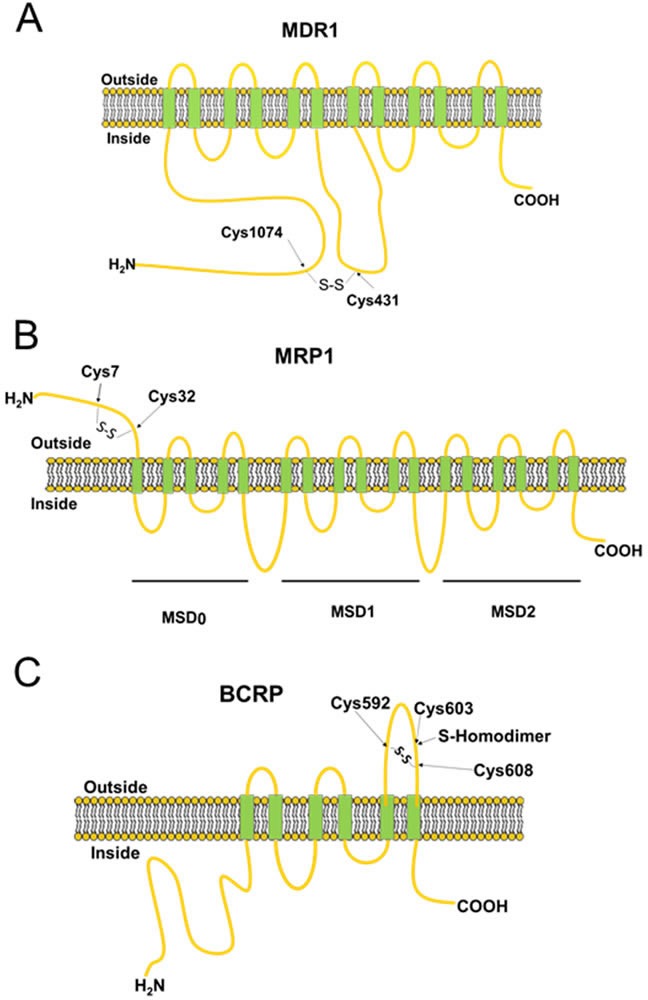
Schematic diagrams showing the structures of MDR1, MRP1 and BCRP All ABC transporters contain transmembrane and membrane-spanning domains. The disulfide bonds between the cysteine residues identified in the figure are required for maintenance of protein stability and transporter function.

All ABC transporters contain four domains - two nucleotide-binding domains (NBDs) and two transmembrane domains (TMDs) (Figure 3) [15]. These four domains can be fused into multi-domain polypeptides in a variety of ways. The driving force for drug transport is achieved by a switch between two principal conformations of the NBD dimer [16]. The conformations of ABC transporters are maintained by multiple chemical interactions, including covalent bonds—the intra- and inter-molecular disulfide bond formed between reactive cysteine residues [17]. The cellular redox status has a great impact on reversible disulfide bond formation and is essential for proper protein folding as well as transporter functions.

The drug transport activity of human MDR1 is correlated with the redox states of its two cysteine residues (Cys431 and Cys1074). The ATP hydrolysis activity is strongly inhibited by the covalent reaction of either of these two cysteine residues with N-ethylmaleimide (NEM), a sulfhydryl blocker [18]. These two cysteine residues are present in NBD1 and NBD2 (Figure 3A), and are located very close to the bound nucleotide. The ready formation of the intramolecular disulfide between Cys431 and Cys1074 shows that the two nucleotide-binding sites of MDR1 are structurally very close and capable of intimate functional interactions, consistent with our current understanding of the catalytic mechanism [19].

MRP1 has a topological configuration similar to MDR1, whereas MRP1 has an additional membrane-spanning domain located at the N-terminus, called MSD_0_ [20]. The MSD_0_ functions as a plug that controls gating during drug transport (Figure 3B) [21]. Mutations at certain cysteine residues within MSD_0_ drastically reduce drug-transport activities [22, 23]. A previous study has identified that MSD_0_ is necessary for the dimerization of MRP1, which can be disrupted by treatment with dithiothreitol (DTT), a reducing agent [24, 25]. These data suggest that dimerization is formed through disulfide linkage between cysteine residues. Yang *et al* [23] investigated the roles of Cys7 and Cys32, which are located in the MSD_0_ domain, in MRP1 dimerization (Figure 3B). Mutations at Cys7 caused conformational changes and prevented dimerization in MRP1 [26]. In addition to dimerization, cancer cells activate antioxidant systems after treatment of ROS-inducing anticancer drugs, including enhanced expression of glutathione (GSH), which can form glutathione S-conjugated molecules to facilitate drug efflux by MRP1 [27].

In contrast to the molecular structures of MDR1 and MRP1, BCRP comprises six transmembrane domains and only one ATP-binding cassette, and is known as a ‘half-transporter’ [28]. Human BCRP exists in the plasma membrane as a homodimer due to disulfide-bonded cysteine residues (Figure 3C) [29]. Treatment with 2-mercaptoethanol (2-ME) reduces the BCRP from homodimer to monomer [30]. Three of the cysteine residues, Cys592, Cys 603, and Cys608 in BCRP are located on the extracellular face between TMD5 and TMD6 (Figure 3C) [31–33]. Cys592 and Cys608 are critical for protein stability by intramolecular disulfide bond formation. Mutations at these two cysteine residues result in protein misfolding and degradation, thereby increasing drug sensitivity because of inefficient drug elimination [31–33]. Cys603 is implicated in intermolecular disulfide bond formation, resulting in dimerization of BCRP (Figure 3C). Mutation at Cys603 prevents homodimerization [33]. However, functional analyses demonstrates that mutation at Cys603 do not change the transport activity of the drugs SN-38 and mitoxantrone, even though monomeric BCRP represents only a half-molecule of a functional ABC transporter [32]. Recently, Cys284, Cys374, and Cys438 are also reported to be involved in intramolecular disulfide bond formation and necessary for BCRP function [34].

### Redox determine transporter gene expression

Apart from the conformational changes of those drug efflux pumps mentioned above, redox-induced overexpression of efflux pumps provides alternative ‘gates’ by which drugs can be exported from cells. Overexpressed transporters have been frequently observed in many types of human malignancy, and correlated with reduced response to chemotherapeutic agents [35]. After treatment with anticancer drugs, redox signaling networks are activated to regulate these transporters expression in multiple layers, including transcriptional, translational, post-translational, and epigenetic levels.

#### Transcriptional regulation

**Figure 4:**
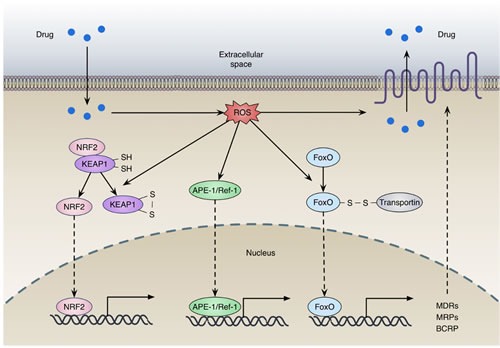
Redox regulation of drug efflux transporters expression (a) Oxidation of KEAP1 dissociates NRF2 from the complex, allowing the translocation and activation of NRF2; (b) Oxidative stress promotes the translocation of APE-1, facilitating transcription of numerous gene including MDRs, MRPs and BCRP; (c) FOXO can be activated by interacting with transportin through disulfide linkage under oxidative stress. The activation of these transcription factors contributes to the expression of drug efflux transporters.

Accumulating evidence shows that redox-sensing transcription factors take part in the transcriptional regulation of drug efflux transporters (Figure 4). Nuclear factor-erythroid 2 related factor 2 (NRF2), a redox-sensing transcription factor, can bind to antioxidant response element (ARE) and regulates a broad spectrum of genes involved in redox balancing, glutathione synthesis, and drug detoxification [36]. AREs are identified in the promoter region of efflux transporters, such as BCRP and MRPs [36]. In general, NRF2 is anchored in the cytoplasm by Kelch-like ECH-associated protein 1 (KEAP1), which facilitates NRF2 ubiquitination and proteasomal degradation. Cys273 and Cys288 of KEAP1 are the crucial target residues for oxidation. Redox modifications dissociate KEAP1 from NRF2, allowing the translocation of NRF2 to the nucleus, where it transactivates target gene expression (Figure 4) [37]. Recent studies showed that higher levels of NRF2 could promote tumorigenesis and contribute to chemoresistance, suggesting a “dark side” of the NRF2 pathway [38–43]. For example, the expression of NRF2 is increased during acquired resistance to tamoxifen and doxorubicin in breast and ovarian cancer cells [44, 45]. Nuclear accumulation of NRF2 can lead to enhanced expression of ARE-containing genes including drug efflux transporters, which facilitate the development of drug resistance [46]. In addition, overexpression of NRF2 causes enhanced resistance to chemotherapeutic agents, including cisplatin, doxorubicin and etoposide [40]. Higher expressions of NRF2 and its target genes are associated with taxol resistance and anchorage-independent growth in MCF-7 and MDA-MB-231 mammospheres compared to adherent cells [47]. Moreover, transport activities of several MRPs are activated by γ-glutamylcysteine synthetase (γ-GCS, the rate-limiting enzyme for GSH *de novo* biosynthesis), which can be induced by NRF2 [48].

Forkhead box O (FOXO) proteins, a family of transcription factors, are deregulated in several cancers including prostate, breast, glioblastoma, rhabdomyosarcoma, and leukemia [49]. As inactivation of FOXOs has been determined to be a crucial step in carcinogenesis, increasing their activity could be a potential therapeutic strategy for cancer treatment [49, 50]. FOXOs are not only responsible for the initial therapeutic response to anticancer drugs, but also involved in the acquisition of drug resistance (Figure 4) [51, 52]. Under continuous stress induced by anticancer drugs, FOXOs can elicit the expression of relevant genes for drug efflux and antioxidant defense, such as MDR1, MRP2, Mn-SOD and catalase [50, 53–55]. For instance, FOXO3 and FOXO1 can induce MDR1 expression in adriamycin-resistant breast cancer cells and K562 leukemic cells [50, 54]. In addition, the promoter region of the human MRP2 gene contains four FOXO binding sites, and transcription of MRP2 gene is stimulated by overexpressed FOXO1 in MCF-7 cells [53]. FOXO1 expression is significantly upregulated in a paclitaxel resistant cells and further enhanced by exposure to paclitaxel [56]. Furthermore, FOXO1 overexpression has been frequently observed in cancer tissue samples obtained from chemoresistant patients [57]. Paradoxically, recent studies showed that FOXO3 expression levels were decreased in cisplatin-resistant cells [58], and FOXO3 knockdown increased cell proliferation and enhanced resistance to cisplatin [59].

Ataxia telangiectasia mutated (ATM) is a serine/threonine protein kinase that participates in activation of the DNA damage checkpoint, resulting in cell cycle arrest, DNA repair or apoptosis [60]. Recent studies have revealed a novel mechanism of ATM activation via direct oxidation [61, 62]. When ATM is activated by double-strand breaks (DSBs), the protein undergoes monomerization that requires free DNA ends and the Mre11-Rad50-Nbs1 (MRN) complex. By contrast, when ATM is activated by direct oxidation, oxidized ATM forms an active dimer covalently linked by intermolecular disulfide bonds [61]. Residue Cys2991 is crucial for ATM activation by oxidation. A C2991L mutant cannot be activated by H_2_O_2_ but can be normally activated by the MRN complex and DNA [61]. A recent study showed that both camptothecin and cisplatin treatment not only induced ATM activation, but also upregulated MDR-related genes BCRP and MRP2 expression in NCI-H446 cells. Moreover, cisplatin and camptothecin-induced BCRP and MRP2 upregulation can be suppressed by ATM inhibitors, indicating the role of ATM activation on MDR formation in lung cancer chemotherapy [63].

#### Post-translational regulation

MDR1 is a phosphorylation substrate for a number of protein kinases, including protein kinase C (PKC) and protein kinase A (PKA) [64]. PKA is shown to be activated by redox modifications through the formation of intramolecular disulfide bonds which cause a subcellular translocation, resulting in phosphorylation of established protein substrates [65]. PKC catalytic properties can be altered by redox mechanisms, which in turn influence the activity of MDR1 [66]. Activation of PKC has been reported to increase the phosphorylation of MDR1 in multidrug-resistant cells [67] and decrease drug accumulation and sensitivity [68]. Conversely, treatment with PKC inhibitors has been shown to decrease the phosphorylation of MDR1, resulting in attenuated drug efflux activity and MDR1 drug binding [69].

#### Epigenetic regulation

The promoter region of MDR1 is highly GC-rich and contains several CpG islands that are prone to be methylated for transcriptional silencing. Studies have demonstrates that the methylation status of the MDR1 promoter is correlated with MDR1 gene transcriptional activity [70–72]. The methylation is catalyzed by DNA methyltransferases (DNMTs) and use of S-adenosylmethionine (SAM) as a methyl donor. SAM is the first metabolite in the methionine cycle catalyzed by S-adenosylmethionine synthetase (also known as methionine adenosyltransferase, MAT). The activities of MATs are profoundly correlated with redox conditions, through the maintenance of a homotetrameric conformation [73]. The methionine cycle is the primary source of cysteine, a precursor of GSH in the transsulfuration pathway. Intracellular GSH levels are essential in the maintenance of methylated DNA. GSH depletion by hepatotoxin bromobenzene results in a reduction of intracellular methionine pools and genome-wide DNA hypomethylation [74].

## ALTERED DRUG METABOLISM

Besides increased rates of drug efflux, altered drug metabolism is another important resistance mechanism, including drug inactivation or deficient drug activation. The redox resetting induced by anticancer drugs may hinder the therapeutic effects by such mechanisms. Antioxidant systems can directly inhibit the antitumor activity of some anticancer agents, such as paclitaxel [75], bortezomib [76] and radiation therapy [77]. For example, buthionine sulphoximine (BSO) significantly increases paclitaxel cytotoxicity through ROS accumulation [75]. Also, platinum drugs, which generate extremely high ROS levels, can be inactivated by GSH [78].

Alternatively, the cellular redox state is correlated with enzymic expression required for the conversion of antimetabolites, such as 5 fluorouracil (5-FU) and methotrexate, to their most active forms [79, 80]. Capecitabine is a fluoropyrimidine prodrug that is converted into 5-FU by thymidinephosphorylase [81]. The gene encoding thymidinephosphorylase can be inactivated by DNA methylation, thereby causing capecitabine resistance [82]. These epigenetic alterations have been shown to be induced by H_2_O_2_, where DNMT1 binds more tightly to chromatin after H_2_O_2_ treatment and then alters the methylation status of CpG regions [83]. As observed in the case of the topoisomerase inhibitor irinotecan, the inactivation by UDP glucuronosyl transferase 1 (UGT1A1) is induced by the redox-sensing NRF2-KEAP1 pathway [84]. Epigenetic silencing can also promote drug activity, and the expression of UGT1A1 is reduced by DNA methylation of the promoter. Therefore, in this case, promoter methylation promotes irinotecan activity [85, 86].

## ALTERATIONS IN THE DRUG TARGETS

Drug response and resistance are also determined by alterations in the drug target, such as mutations or changes in expression level. The deregulated or prolonged production of cellular oxidants has been linked to mutations (induced by oxidant-mediated DNA damage), as well as modification of gene expression [87]. Thus, target alteration is more likely to happen with anticancer drugs that induce high ROS levels.

**Figure 5:**
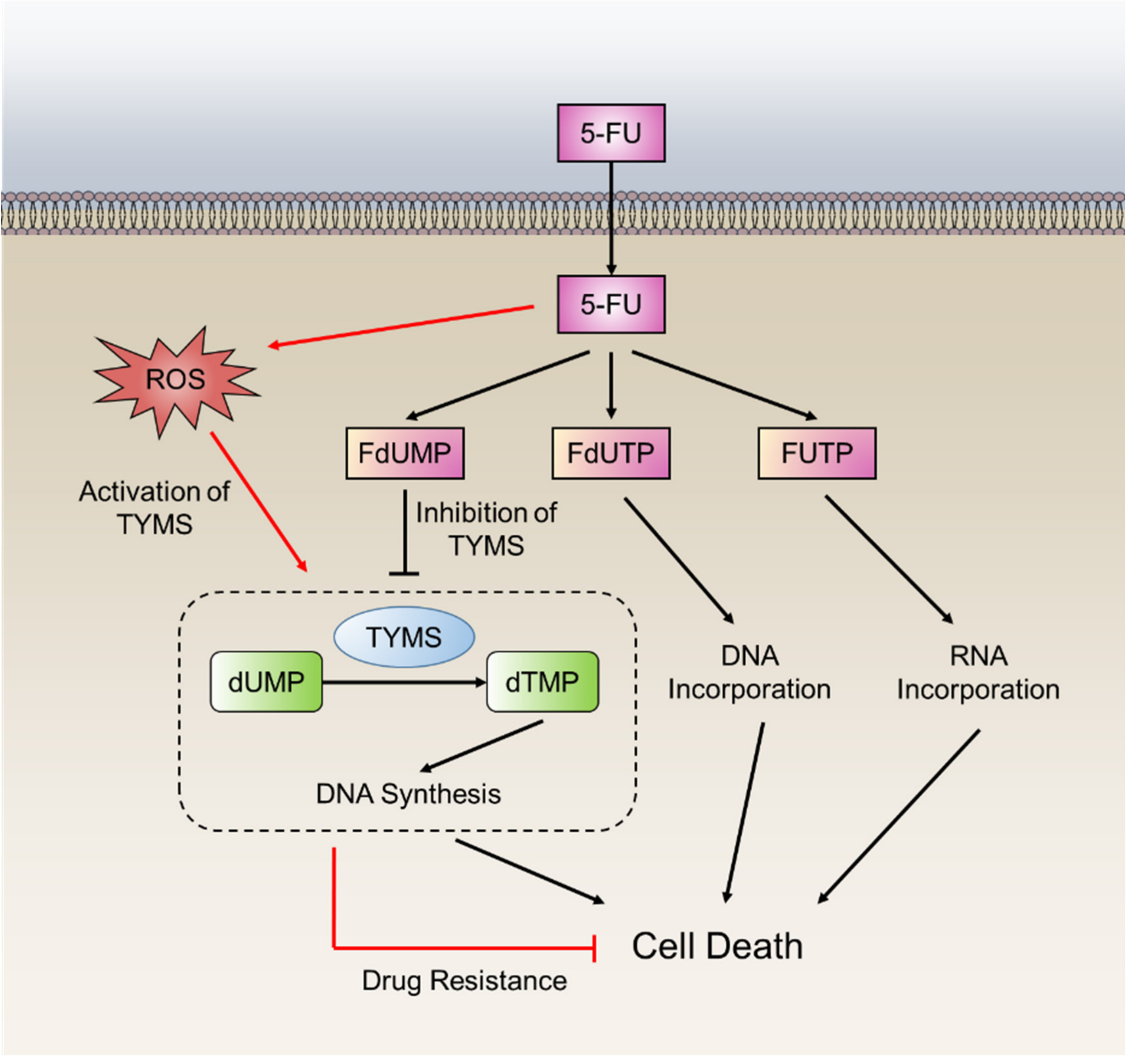
5-FU resistance in cancer cells by TYMS oxidation The fluoropyrimidines (5-FU) are broken down into three metabolites, fluorodeoxyuridine monophosphate (FdUMP), fluoro-deoxyuridine triphosphate (FdUTP) and fluorouridine triphosphate (FUTP). The principal mechanism of action of 5-FU is the inhibition of thymidylate synthase (TYMS), but alternative pharmacodynamic pathways acting through incorporation of drug metabolites into DNA and RNA. TYMS can also be activated through direct oxidation that leads to 5-FU resistance.

The fluoropyrimidine 5-FU is widely used in the treatment of a variety of cancers, including colorectal, breast, and aerodigestive tract cancer [88]. It is converted intracellularly to three active metabolites: fluorodeoxyuridinemonophosphate (FdUMP), fluorodeoxyuridinetriphosphate (FdUTP) and fluorouridine triphosphate(FUTP) (Figure 5). These active metabolites disrupt RNA synthesis and the function of thymidylate synthase (TYMS). TYMS plays a crucial role in catalyzing deoxyuridylate (dUMP) to thymidylate (dTMP), which provides the sole intracellular *de novo* source of dTMP [89]. Human TYMS protein can specifically bind to its own TYMS mRNA and functions as a translational repressor. The RNA binding activity is determined by its redox state. In the presence of reducing agents, the RNA binding activity of TYMS protein is significantly enhanced. In contrast, treatment of TYMS protein with the oxidizing agent diamide inhibits RNA binding [90]. These results demonstrate that the oxidation of TYMS, resulting in loss of translational repressor function, could lead to 5-FU resistance in cancer cells.

Drug target changes through epigenetic events have also been shown to be involved in resistance to cytotoxic chemotherapy drugs in a range of tumor cells. Hypermethylation of the DNA promoter regions of the drug targets results in cell resistance to anticancer drugs, such as cisplatin and carboplatin [91, 92]. In addition, methylation of genes involved in apoptosis, including the DNA mismatch repair (MMR) gene human mutL homolog 1 (hMLH1), can occur in drug-resistant tumor models. This has led to the concept that the use of a DNA demethylating agent such as 2′-deoxy-5-azacytidine in combination with anticancer drugs may reverse this resistance mechanism [93].

## INEFFECTIVE INDUCTION OF CELL DEATH

Following the action of an activated drug on its cellular target, the therapeutic outcome is then determined by the next key process; the response of cancer cells to drug treatment. Generally, oxidative stress causes by anticancer drugs in turn leads to some cellular damage (e.g., DNA damage) that is tightly coupled to the induction of cell death. Nevertheless, some intrinsic redox adaptive responses can be triggered to enable the cancer cells to survive through inhibition of cell death and activation of cellular survival pathways, thus providing a mechanism of resistance to treatment with anticancer agents [7].

### Deregulation of apoptosis

**Figure 6:**
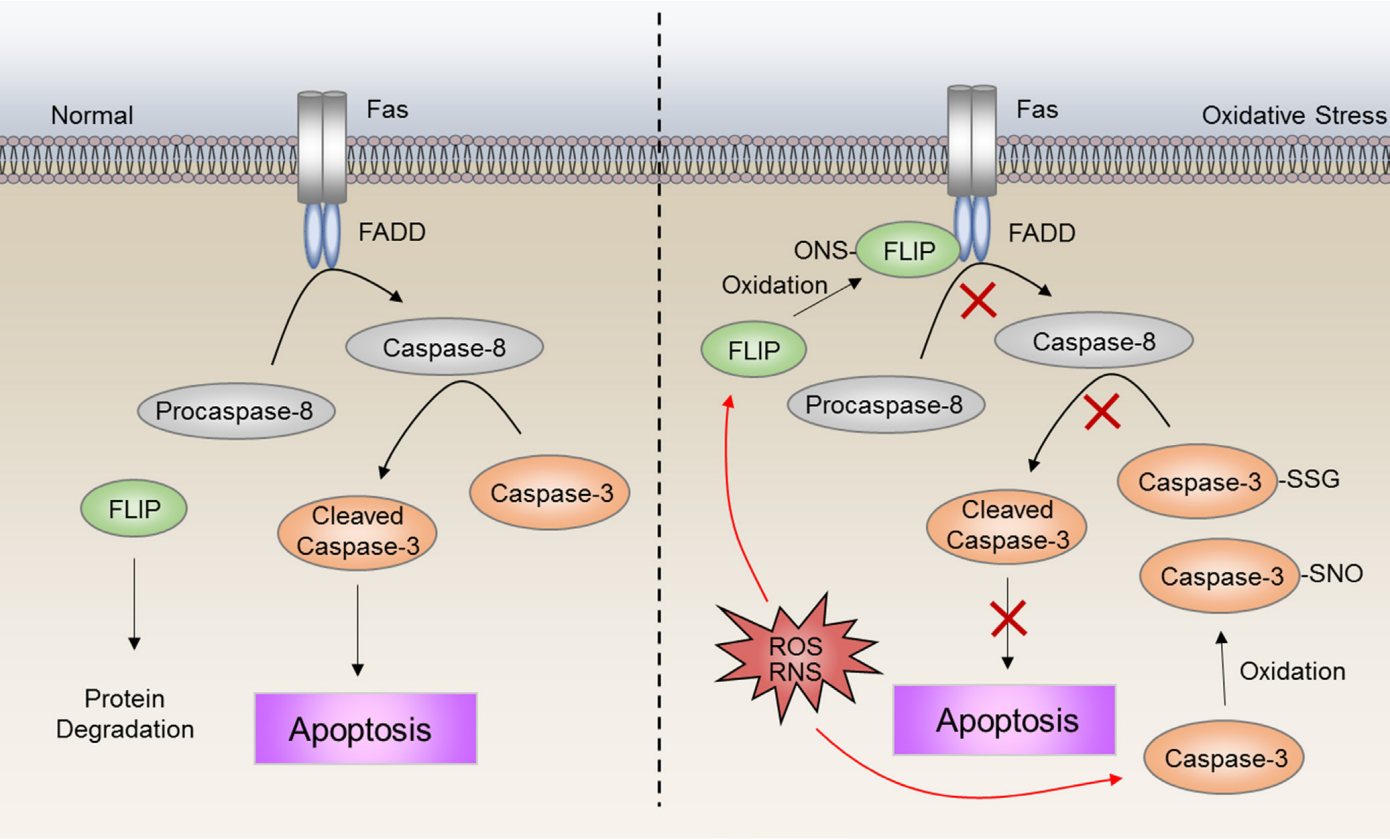
ROS-induced deregulation of apoptosis S-nitrosylation of FLIP inhibits the interaction between procaspase-8 and FADD, leading to inactivation of caspase-8. S-glutathionylation and S-nitrosylation of caspase-3 inhibit cleavage to the active form. The high ROS level in drug-resistant cells may contribute to escape from apoptosis by S-nitrosylation of FLIP, as well as S-glutathionylation and S-nitrosylation of caspase-3.

It is well known that resistance to apoptosis is a hallmark of cancer [94]. Thus, deregulation of apoptosis will protect cancer cells from cell death caused by drug-induced cellular damage. Cleavage of caspase-3 is known to play a central role in apoptosis. Substantial evidence reveals that the activity of caspase-3 is inhibited *via* redox modifications [95]. Caspase-3 has been found to be constitutively S-glutathionylated in human umbilical vein endothelial cells (HUVECs) [96]. Upon tumor necrosis factor α (TNFα) stimulation, de-glutathionylation of caspase-3 occurs mediated by glutaredoxin (Grx). Knockdown of Grx notably inhibit TNFα-induced cell death owing to attenuated caspase-3 cleavage, concomitant with enhanced caspase-3 S-glutathionylation [96]. Mutations of key S-glutathionylation sites of caspase-3 (C163S, C184S, and C220S) enhance cleavage compared with wild-type caspase-3 [97]. Furthermore, S-glutathionylated caspase-3 inhibits its cleavage by caspase-8 *in vitro* (Figure 6) [97]. In addition, caspase-3 can also be S-nitrosylated at Cys163 [98]. Upon the first apoptosis signal (Fas) ligation, de-nitrosylated caspase-3 leads to caspase-3 activation (Figure 6) [99]. Collectively, the higher ROS levels in drug-resistant cells may contribute to their escape from apoptosis by caspase-3 S-glutathionylation and S-nitrosylation.

Upon Fas ligand (FasL) binding, Fas interacts with Fas-associated protein with death domain (FADD) and procaspase 8 or 10, to form an active death inducing signaling complex (DISC) [100]. The FADD and procaspase-8 interaction can be inhibited by Flice inhibitory protein (FLIP) through competitive binding to FADD [100, 101]. Intriguingly, the activity of FLIP is shown to be enhanced by S-nitrosylation [102]. Loss of S-nitrosylation increases FLIP degradation, which in turn facilitates DISC complex formation, and results in activation of the downstream apoptosis cascade (Figure 6) [102]. FLIP have been shown to be involved in cisplatin-resistance to bladder cancer cells [103]. Also, fibroblast growth factor receptor 4 (FGFR4) has been indicated to be an inducer of chemoresistance in colorectal cancer through regulation of FLIP expression [104]. Thus, inhibition of FLIP may be a promising therapeutic strategy in numerous drug-resistant cancer scenarios [105]. Taken together, these studies described herein highlight that apoptosis is deregulated at multiple layers *via* redox-associated mechanisms.

### Activation of autophagy

Autophagy plays paradoxical roles in acquired resistance to anticancer drugs. On one hand, cytotoxic drug treatment triggers persistent autophagy, which will produce excessive cellular damage and even lead to cell death, thus attenuating the drug resistance activity of cancer cells [106]. On the other hand, autophagy has a role in maintaining cancer cell survival during conditions of stress and might mediate resistance to anticancer therapies [107, 108]. For example, co-administration of cisplatin and an autophagy inhibitor chloroquine significantly suppress tumor survival whereas cisplatin monotherapy fails to show anticancer activity in nude mice xenografts using EC109/CDDP cells [109]. Another study demonstrated that in chronic lymphocytic leukemia (CLL), autophagy was induced by multiple stimuli and acted as a mechanism of resistance against flavopiridol, an endoplasmic reticulum (ER)-stress-mediating agent [110].

Redox resetting has been shown to regulate autophagy at multiple levels. To start with, high cellular ROS accumulation has been widely proven to induce autophagy [111]. In response to ER stress induced by tunicamycin, but not thapsigargin, NADPH oxidase 4 (Nox4)-mediated production of H_2_O_2_ leads to cytoprotective autophagy in HUVEC cells [112]. Treatment with either the antioxidant N-acetyl-L-cysteine (NAC) or catalase hinder the conversion of LC3-I to LC3-II which is a key step in autophagy induction, thereby decreasing the formation of LC3-II positive autophagosomes, and reducing starvation-induced protein degradation [113]. The activity of Atg4, which has been shown to be involved in the processing of LC3, has also been proved to be sensitive to H_2_O_2_ [113]. However, antioxidant activity is also essential for autophagy induction. For example, overexpression of catalase increases LC3-II levels in both HCT116 cells and H460 cells with low levels of endogenous catalase [114, 115]. Inhibition or knockdown of catalase attenuates LC3-II accumulation in HCT116, H1299 as well as WI38 cells [114, 115]. Due to the paradoxical roles autophagy plays in cancers, a better understanding of how redox regulation distinguishes between the survival-supporting and death-promoting roles of autophagy is necessary [116].

### DNA damage repair

The anticancer activity of most chemotherapy drugs relies on the induction of DNA damage in rapidly cycling tumor cells with inadequate DNA repair [117]. The cellular response to DNA damage is either repair or cell death. Therefore, the DNA damage repair capacity of cancer cells has a significant influence on the efficacy of DNA-damaging drugs.

The redox environment is capable of directly modulating DNA repair. One of the initial pieces of evidence is that both an increase in 8-oxoguanine (8-oxoG) and a reduction in DNA repair occurs *in vitro* following treatment with cadmium [118]. This is subsequently shown to be due to cysteine modification of 8-oxoguanine DNA glycosylase 1 (OGG1) [119]. Furthermore, an interaction between OGG1 and poly (ADP-ribose) polymerase 1 (PARP-1), a sensor of DNA damage involved in DNA repair, has recently been described [120]. This interaction is enhanced by oxidative stress and can stimulate PARP-1 activity [120]. Oxidative stress also causes the translocation of the Y-box binding protein (YB-1) to the nucleus, where it has a stable interaction with nei-like 2 protein (NEIL2) and increases NEIL2 activity in the base excision repair (BER) pathway [121]. A further example of redox regulation of DNA damage repair is the interaction between oxidized XRCC1 (x-ray cross-complementing group 1) and DNA polymerase b (Pol b) which is enhanced due to the formation of a disulfide bond [122].

Apurinic-apyrimidinic endonuclease 1 (APE-1) is a versatile protein that has both DNA repair and transcriptional regulatory functions by facilitating transcription factors binding to DNA [123, 124]. Overexpression of APE-1 has been found in several cancers and are correlated with the tumor radiosensitivity [125]. For example, APE-1 contributes to radioresistance [126] and alkylating agent resistance [127] in human glioma cells, as well as also promotes resistance to radiation combined with chemotherapy in medulloblastoma, primitive neuroectodermal tumors and pediatric ependymomas [128]. Knockdown of APE-1 dramatically sensitizes cancer cells to radiotherapy in pancreatic carcinoma [129]. APE-1 can be activated by nontoxic levels of ROS that promote translocation into the nucleus (Figure 4) [130]. ROS production following Ca^2+^ mobilization *via* purinergic receptors-induced extracellular ATP stimulation is responsible for the localization of APE-1 [131]. Furthermore APE-1 phosphorylation by PKC after an oxidative challenge has been shown to increase the activity of the APE-1 redox domain [132].

In addition, the activities of other DNA-repair proteins such as Ku, ATM, and human replication protein A (RPA) have been also reported to be altered by the redox resetting. In the nonhomologous end joining (NHEJ) double-strand DNA repair pathway, Ku DNA binding is lower in an oxidizing environment, although the mechanism is not clear [133]. Ku is a heterodimer that encircles broken DNA ends during repair and can recruit the DNA-PK catalytic subunit (DNA-PKcs) [134]. The duration of binding of Ku to the DNA is needed to improve recruitment of DNA-PKcs to the DNA-PK complex [135]. Ku is inactivated during oxidative stress in glucose-6-phosphate dehydrogenase (G6PD) null mutant Chinese hamster ovary cells [136]. ATM is subsequently activated through oxidation at specific cysteine residues [61]. Evidence also shows that ATM can promote an antioxidant response *via* regulation of the pentose phosphate pathway—one of the primary sources of NADPH [137]. An additional example of a DNA repair pathway protein involved in oxidative stress is human RPA. RPA is a DNA-binding protein involved in replication, repair, and recombination. In an oxidizing environment, the cysteines in the zinc-finger motif of the p70 subunit form disulfide bonds that impair its DNA binding [138].

It has been established that the detection of MMR is associated with resistance to many, but not all, DNA-damaging anticancer agents, such as monofunctional alkylating agents, cisplatin, and the antimetabolite 6-thioguanine [139]. Alkylating agents, including the chloroethylnitrosoureas (carmustine [BCNU], lomustine, and fotemustine), temozolomide, and procarbazine, are commonly used for the treatment of malignant brain tumors [140]. These agents add alkyl groups to DNA causing DNA damage and apoptosis [141]. Resistance to alkylating agents through direct DNA repair by O6-methylguanine methyltransferase (MGMT) remains a significant barrier to successful treatments of patients with malignant glioma [142, 143]. The relative expressions of MGMT in tumor cells may determine the response to alkylating agents [141]. Moreover, promoter methylation can silence MGMT expression in gliomas [144]. Recent studies showed that oxidative damage induced the formation of a large complex containing the DNMTs and polycomb repressive complex 4 (PRC4) members, which could lead to MGMT promoter methylation [145]. Early clinical studies showed that glioma patients with methylated MGMT promoters had a survival benefit treated with radiotherapy [146].

## TARGETING REDOX ALTERATIONS IN CANCER THERAPY

In general, cancer cells exhibit higher levels of ROS than normal cells that facilitate tumorigenesis and tumor progression. Therefore, the treatment of antioxidants can suppress cancer initiation or progression. A number of studies suggest that antioxidants could diminish cancer initiation by suppressing DNA damage and genomic instability. For example, ATM-deficiency-accelerated transgenic murine lymphomagenesis is suppressible by NAC [147]. Another study has also claimed that NAC slowed tumor progression in a p53-dependent mouse lymphomagenesis model, seemingly by reducing genomic instability [148]. Furthermore, a recent study has also observed that NAC and vitamin C have significant antitumorigenic effects *in vivo*. But in stark contrast to earlier studies, they found that the effect of NAC and vitamin C highly relied on hypoxia-inducible factor 1 (HIF-1) rather than on reduction of genomic instability in a MYC-dependent lymphoma model [149]. However, other studies showed that supplementation with vitamin E significantly increased the risk of prostate cancer [150] and taking b-carotene, vitamin A or E supplements increased the incidence of lung cancer [151]. A recent study showing that NAC promotes melanoma progression supports these findings [152]. Likewise, a study showed that NAC increased melanoma metastasis *in vivo* through the small guanosine triphosphatase (GTPase) RHOA activation [153].

Based on the intrinsic oxidative stress of cancer cells, further ROS inductions have been shown to be efficient in preferentially killing malignant cells, with some showing promise in clinical studies. However, upregulated antioxidant capacity has been found in some cancer cells, especially those in advanced stages. This redox adaptation enables the cancer cells to survive under increased oxidative stress, and provides a mechanism of drug resistance. For example, elevated levels and activity of catalase are found in multidrug resistant HL-60 leukaemia cells [154]. Upregulation of HMOX1, SOD1 and GSH are found to be associated with arsenic trioxide resistance [155]. Also, several studies suggest that the resistance to doxorubicin, paclitaxel or platinum-based drugs, which induce intracellular ROS production, is correlated with increased antioxidant capacity [156, 157].

For those cancer cells that have adapted to higher level of oxidative stress by increasing their antioxidant capacity, simply ROS-generating agents treatment might not be effective. It is possible to combine ROS-generating drugs with compounds that restrain the cellular antioxidant capacity. For example, a combination of arsenic trioxide and 2-Me, a SOD inhibitor, shows significantly enhanced cytotoxic activity in primary chronic lymphocytic leukaemia (CLL) cells [158]. A combination of arsenic trioxide and ascorbic acid, mediating GSH depletion, are also reported to be effective for relapsed or refractory multiple myeloma in a clinical study [159].

## CONCLUSIONS

Due to significant advances in the research arena in the last few decades, cancer drug resistance is now realized to be more complicated than originally conceived. The emergence of drug resistance is the result of dynamic battles between cancer cells and chemotherapeutic agents. Although new anticancer drugs will continue to be developed, it is anticipated that novel drug-resistance mechanisms will follow. Therefore, deciphering the intrinsic mechanisms of drug resistance induction may be an effective strategy to solve this significant problem in cancer therapy.

Redox resetting, which usually occurs in anticancer drug treatment, is a protective response from tumor cells that can buffer drug-induced stresses and damage by rebuilding redox homeostasis and activating multiple redox signaling pathways, thereby leading to drug resistance. The versatility of redox signaling is such that it can affect almost every cell and involve multiple signaling processes. Thus it is anticipated that redox signaling will continue to be an important stimulator in the development of drug resistance with new therapeutic agents. Therefore, it is essential to reveal and understand the molecular mechanisms underlying redox resetting-induced drug resistance.

Despite the universality of redox resetting in chemotherapeutic treatments, different agents for distinct cancer types, patients with individual variations, and genetic heterogeneity in tumors may lead to inequable situations. Therefore, only when we have screened and identified enough usable drug-resistant biomarkers related to redox resetting, will it be feasible to overcome drug resistance by monitoring and regulating the process of redox resetting. The use of modern genomic, proteomic and other omics techniques has dramatically promoted the ability to identify novel genes and signaling networks involved in tumor responsiveness to drug treatment. Moreover, high-throughput techniques combined with bioinformatics approaches has allowed the identification of genotypes and molecular signatures also aided the interrogation of clinical samples, facilitating the prediction of drug responses to certain drugs. These will provide abundant data that can be used to identify potential predictive biomarkers for patient stratification.

## Abbreviations

ROS: Reactive oxygen species
ABC: the ATP-binding cassette
MDR: multidrug resistance
MRP: MDR-associated protein
BCRP: breast cancer resistance protein
NRF2: Nuclear factor-erythroid 2 related factor 2
ARE: antioxidant response element
FOXO: Forkhead box O
ATM: Ataxia telangiectasia mutated
DNMTs: DNA methyltransferases
5-FU: 5 fluorouracil
APE-1: Apurinic-apyrimidinic endonuclease 1
